# Epidemiological and immunopathological studies on *Porcine parvovirus* infection in Punjab

**DOI:** 10.14202/vetworld.2016.827-831

**Published:** 2016-08-08

**Authors:** Amninder Kaur, V. Mahajan, G. D. Leishangthem, N. D. Singh, Payal Bhat, H. S. Banga, G. Filia

**Affiliations:** 1 Department of Veterinary Pathology, Guru Angad Dev Veterinary and Animal Sciences University, Ludhiana - 141 004, Punjab, India; 2 Animal Disease Research Centre, Guru Angad Dev Veterinary and Animal Sciences University, Ludhiana - 141 004, Punjab, India

**Keywords:** abortion, enzyme-linked immunosorbent assay, histopathology, immunohistochemistry, indirect, *Porcine parvovirus*, swine

## Abstract

**Aim:**

The aim of this study was to get the first-hand knowledge about the seroprevalence of *Porcine parvovirus* (PPV) in Punjab and a diagnosis of PPV from abortion cases of swine using gross, histopathological, and immunohistopathological techniques to observe the tissue tropism of the virus strain.

**Materials and Methods:**

Tissue samples from the reproductive tract of pig (n=32), placental tissue (n=10), and aborted fetuses (n=18) were collected from Postmortem Hall of the Department of Veterinary Pathology, GADVASU, field outbreaks and from butcher houses in and around Ludhiana. These samples were processed for histopathological and immunohistochemical (IHC) studies. For seroprevalence study, 90 serum samples of different sex and age were collected from 15 swine farms of Punjab and were subjected to indirect enzyme linked immunosorbent assay using commercial kit.

**Results:**

Overall, seroprevalence of PPV was found to be 41.1%. Sex and age related difference in the prevalence was noted. In abortion cases grossly congested and emphysematous lungs, congested internal organs with fluid in abdominal cavity and congestion in brain, changes were noted in fetuses, while diffuse hemorrhages and edema was observed in placental tissue. Histopathologically, the most frequent fetal lesions in aborted fetuses were noted in lungs, liver, and brain. IHC staining revealed PPV antigens in sections of heart, liver, lung, spleen, brain, lymph node of fetuses, placenta, and uterus of sow. Gross, histopathological, and IHC examination of the samples confirmed 5 fetus, 2 placenta and 3 female reproductive samples positive for parvovirus infection.

**Conclusions:**

Seroprevalence results may serve as a support either in prevention or control of the disease. IHC is the sensitive technique for diagnosis of PPV associated with the reproductive tract of swine and was found to supplement the gross and histopathological alterations, respectively, associated with the disease.

## Introduction

Commercial pig farming is one of the best and profitable businesses in India. As per present scenario, pork production in India represents only 7% of the country’s animal protein sources and its population is 10.29 billion in the country according to 19^th^ livestock census. According to 18^th^ livestock census, Punjab contributed 0.23% pigs toward the total livestock population within the country which has increased by 42% in recent years. In Punjab, pork production is 500 MT (million ton), which is 0.21% of country’s pork production. It is predicted that pork consumption will be doubled in next decade. However, diseases of the reproductive tract of swine are a major constrain in this regard as it cause a huge economic loss to the pig farmers in the form of death of the fetus and infertility in sows. Reproductive problems are the 3^rd^ major cause of swine mortality [1]. *Porcine parvovirus* (PPV) is considered to be one of the major causes of reproductive failure in swine characterized by the repeat of estrus, abortion(s), and the delivery of mummified or stillborn fetuses [2]. The virus is endemic in most areas of the world and can be found in all pig herd categories [3].

PPV is the ubiquitous infectious cause of reproductive failure in swine worldwide [4]. PPV is a small non-enveloped, single-stranded DNA virus which is classified in the genus parvovirus (Latin *parvus*=small) of the family *Parvoviridae* [5]. Although there are various viral pathogens of swine which affect its reproductive performance include porcine reproductive and respiratory syndrome virus (PRRSV), PPV, *Porcine circovirus* Type 2 (PCV2), classical swine fever virus, auzesky disease virus, and porcine enterovirus, PPV is considered to be one of the major causes of reproductive failure in swine characterized by the repeat of estrus, abortion(s) and the delivery of mummified or stillborn fetuses. The virus is transmitted to a new herd either by oronasally, transplacentally, clothing of farmers, and rodents [3]. Boars may play a significant role in the dissemination of PPV by semen [6]. Reproductive failure due to PPV is characterized by a combination of resorption, mummification, and stillbirths in a single litter [4]. PPV infections are commonly described with the acronym stillbirth, mummification, embryonic death and infertility. The virus is endemic in most areas of the world and can be found in all pig herd categories [3,4]. Various serological tests, *viz*., hemagglutination inhibition (HI), immunodiffusion test, virus neutralization test, and ELISA can be used for detection of antibodies against virus in serum samples [7]. ELISA is an effective and more reliable method to detect PPV antibodies in serum samples [7]. Recent studies suggested that coinfection of PPV with PCV2 or PRRSV could occur resulting in more severe reproductive failure and neonatal mortality [8].

Moreover, till date, no studies regarding PPV are reported from Punjab. Screening of PPV infection has been carried out for the first time. Thus, in this study, a comprehensive diagnosis of PPV was done using indirect enzyme-linked immunosorbent assay (i-ELISA), histopathological and immunohistochemistry (IHC) techniques.

## Materials and Methods

### Ethical approval

The study was duly approved by the Institutional Animal Ethics Committee of Guru Angad Dev Veterinary and Animal Sciences University, Ludhiana, Punjab.

### Seroprevalence studies

A total of 90 adult pigs were randomly selected from 15 swine farms located in 8 districts of Punjab using random number table. Total swine population of these selected farms was 800, out of which 90 (approximately 11%) belonging to various swine owners were randomly selected. The permission of sampling and other procedures was duly approved by Institutional Animal Ethics Committee. About 5 ml of blood was collected from each of the pig aseptically from the ear vein in a test tube. The serum was collected from clotted blood by centrifuging at 3000 rpm for 10 min. The serum was collected and stored at −20°C until they were tested for antibodies to PPV using commercially available indirect ELISA kit (Ingezim, Ingenasa). Samples from animals of different sex and age group, i.e., 73 females (30 gilts and 43 adults) and 17 males were collected.

### Pathological studies

A total of 32 reproductive tract samples (26 female and 6 male), 10 placenta, and 18 aborted fetuses were collected from Postmortem Hall of Department of Veterinary Pathology, GADVASU, field outbreaks and from butcher houses in and around Ludhiana. Among the above collected tissues 10 complete cases were collected, i.e., 10 dams which died and were brought to Post-Mortem Hall, GADVASU and their reproductive tract, placenta and feti were collected. The remaining tissues, i.e., 22 reproductive tracts and 8 aborted fetus were collected from slaughter house and field outbreaks, respectively. Irrespective of the place/case of sampling all the tissues (10 dam cases and others) were collectively categorized under the subhead reproductive tract (n=10+22=32), placenta (10) and fetus (n=10+8=18) as one of the objectives of the study was to observe the tissue trophism of the virus strain. In addition to the above-mentioned tissues lung, heart, liver, spleen, lymph node, kidneys, brain, intestine, etc., were collected in 10% neutral buffered formalin for histopathology and IHC. After fixation in 10% neutral buffered formalin, tissue samples were given overnight washing under tap water. Then, dehydration of samples was done through ascending grades of alcohol followed by clearing with acetone and benzene. Tissues were embedded in paraffin wax (Leica Microsystem, Paraplast tissue embedding medium, 56°C) for further processing and 4-5 µ sections were cut using rotary type microtome. The paraffin sections were stained with routine hematoxylin and eosin technique [9]. Wherever necessary, duplicate serial paraffin tissue sections were also stained with Triple shorr stain as per standard protocol [10].

### IHC studies

The tissue sections showing microscopic lesions pertaining to parvo viral infection were further subjected to IHC for confirmation. For IHC studies 4-5 µ thick paraffin embedded tissue sections were cut and mounted on Superfrost Plus, positively charged microscopic slides. The slides were then kept on hot plate to melt the paraffin at 60°C for 30 min and stored till further use. The antigen retrieval was carried out in citrate buffer (pH 6.0) at 99°C for 3 min and 70°C for 7 min using EZ-Retriever^®^ System (BioGenex Laboratories Inc., San Ramon, California, USA). IHC staining with 1:100 dilution of the primary antibody (VMRD, PPV) in phosphate buffer saline was done using advanced SS™ One-Step Polymer-HRP IHC Detection System (BioGenex Laboratories Inc., San Ramon, California, USA). A duplicate section was stained simultaneously by omitting the primary antibody and was used as negative control.

### Statistical analysis

The mean histopathological scores that represented the overall damage to histo-architecture were calculated and further compared with the IHC score of the respective cases using SPSS software.

## Results and Discussion

### Seroprevalence

Serum samples of these selected animals were analyzed by i-ELISA which revealed the overall prevalence of PPV to be 41.1%. The seroprevalence observed in this study was similar to that described by Roic *et al*. [11] with a seropositivity of 56.3%. High seropositivity of 97% was obtained in ELISA by Tummaruk and Tantilertcharoen [12]. This is the first time that PPV seroprevalence was carried out in Punjab as this being the first study on parvovirus infection in pigs in Punjab. The noted high seroprevalence of the disease can be due to the endemic nature of the virus in pig population or the coinfection of this virus with other reproductive tract agents [8]. The seroprevalence of the disease was non-significantly higher (Chi-square=0.102, p=0.995) in males (41.2%) than females (41.1%). High seropositivity among male swine populations indicates that infected boar can be a route of parvovirus infection to female, as various authors also corroborated the same observations. The previous studies have already reported PPV in semen of naturally infected boars [6]. A non-significantly (Chi-square=0.025, p=0.874) higher percent prevalence of PPV was seen in the adult females (41.9%) than the gilts (40.0%). These results were in accordance with the previous study [13]. A high seropositivity in adult animals may be due to the fact that with increasing age, chances of contracting natural infection increases. However, some gilts escape infection till the first gestation, due to multiple factors.

### Pathological and IHC studies

**Figure-1:**
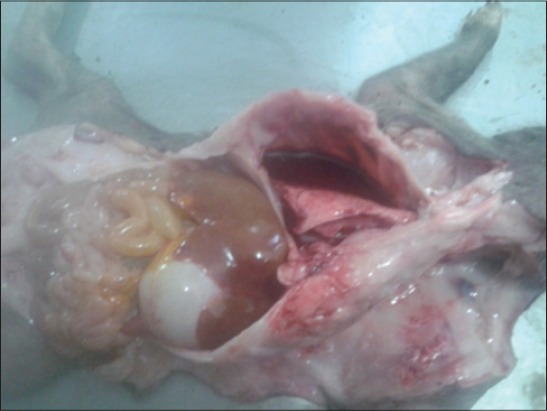
Blood tinged fluid in thoracic cavity of aborted fetus.

About 18 aborted fetuses (stillborn, mummified, dead, and aborted fetuses) were collected from different locations. On post-mortem of these fetus grossly, congested and emphysematous lungs, congested internal organs with fluid in the abdominal cavity (Figure-1) and congestion in brain were the important findings. These findings are in concordance with that of previous studies [14]. Microscopic examination of placenta revealed diffuse hemorrhages and edema. PPV crosses the placenta and attach to the outer surface of the zona pellucida leading to mortality, impaired blastocyst growth and damage of tissues of the maternal reproductive tract [15]. Moreover, death of the conceptus results from the collective damage to tissues and organs and its manifestation depends on the gestational stage at time of infection [2]. Serosanguineous fluid in the body cavity of the fetus is the outcome of damage to circulatory system which indicates the importance of endothelium damage in pathogenesis of parvovirus [16].

**Figure-2:**
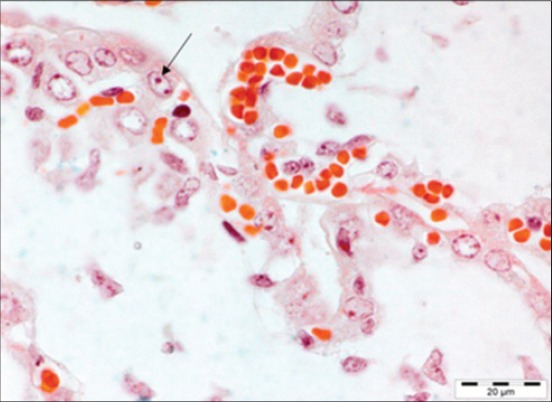
Placenta: Intranuclear inclusion bodies. Triple shorr ×100.

Histopathological examination of different organs of these fetuses revealed necrosis and vacuolar degeneration of hepatocytes, mononuclear cell infiltration in the liver with intranuclear inclusion bodies in hepatocytes, mononuclear cell infiltration in myocardium with mineralization, and fibrosis in aborted fetuses. In addition, congestion in lungs with fibrosis and mononuclear cell infiltration, lymphoid depletion in spleen, submeningeal infiltration of mononuclear cells, gliosis leading to glial nodule formation, congestion and inclusion bodies in cerebellum in the brain and hemorrhage and tubular degeneration in the kidney of fetuses were the notable microscopic observations. These findings are in agreement with that of previous studies [15,16]. Congestion, mononuclear cell infiltration and intranuclear inclusions bodies were also observed in placenta (Figure-2). PPV mainly affects the vasculature of the conceptus and placenta [2]. The histopathological lesions found in infected fetuses may be due to the immune response of the fetus to viral antigen. However, pathological findings reported in the present study may be due to the coinfection with other infectious agents [15].

Histopathological changes suspected to be of viral infection in samples of female reproductive tract (n=26) and male reproductive tract (n=6) of pig were characterized by focal mononuclear cell infiltration in the endometrial layer and inclusion bodies in uterus, cystic ovaries, and hemorrhage in cortex. These microscopic lesions in female reproductive tract may be due to damage of blastocyst layer resulting in damage to the maternal tissue [4]. However, these lesions can be the outcome of coinfection with other infectious agents of the reproductive tract. In this study, no macroscopic or microscopic changes were seen in male reproductive tract as PPV has a little clinical effect on mature boars. Moreover, infected boars become carriers and spread infection to non-infected females [2]. Inclusion bodies seen in the liver of fetus, placenta and uterus of dam was stained specifically with triple shorr stain.

**Figure-3:**
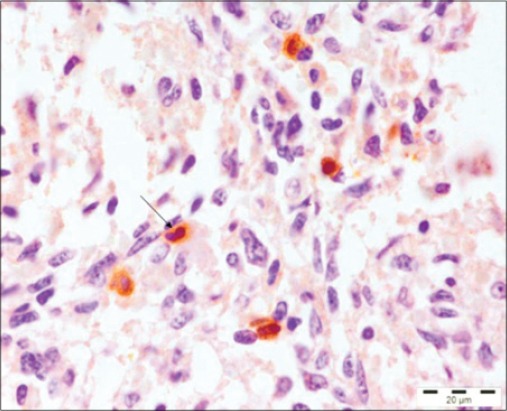
Spleen: Positive immunoreactivity of *Porcine parvovirus* antigen in spleen. Immunohistochemistry, Gill’s hematoxylin counter stain ×100.

**Figure-4:**
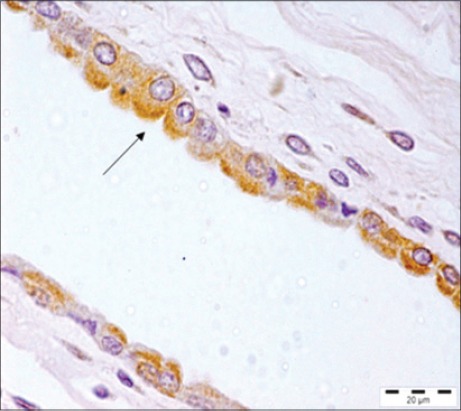
Positive immunoreactivity of *Porcine parvovirus* antigen in placenta. Immunohistochemistry, Gill’s hematoxylin counter stain ×100.

A number of techniques like virus isolation, immunofluorescence, hemagglutination, and IHC can be used to detect PPV antigen in aborted fetuses [17]. IHC has been used as confirmatory diagnostic tools for PPV infection [14,18]. Thus, in this study, IHC has been used for the demonstration/localization of parvovirus antigen in formalin-fixed, paraffin embedded tissues of dams (reproductive tract, placenta) and aborted fetuses to observe the tissue tropism of the PPV. Immunolocalization of PPV antigen was observed in the cytoplasm of the myocytes of heart, hepatocytes of liver, alveolar epithelial cells of the lung, inflammatory cells of the spleen and lymph node of five fetuses (Figure-3). These findings were similar to the findings of the previous study [9] which demonstrated the PPV in lungs of stillborn fetus in coinfection with PCV2. PPV replicates in cells of the monocyte-macrophage series and may lead to impairment of the immune cells [8]. Moreover, the presence of PPV in lymphoid organs of the fetus demonstrated by IHC in the present study was in agreement with the previous literature [3] where it was suggested that after crossing the placenta fetal tissues provide a favorable environment for viral replication. In the placenta of the aborted fetus, PPV antigen was also demonstrated in 2 placenta of aborted fetus (Figure-4). However, the exact mechanism that how the PPV crosses this placental barrier and reaches the fetus is still not understood [2]. In addition, positive immunostaining to PPV was seen in muscular portion of uterus of 3 sows.

Organ wise IHC score was calculated on the basis of antigen positive cells and it showed a high immunoreactivity in the liver of fetus followed by heart and lung. Further, IHC score of the liver of positive fetus was compared with its mean histopathological score and a positive Pearson’s correlation coefficient (r=0.809381) was found between them (Table-1). Similarly, a positive Pearson’s correlation coefficient (r=1.00) was found between histopathological lesions and IHC expression in uterus positive for PPV (Table-2).

**Table-1:** Overall histopathological and IHC scoring of liver of fetus positive for PPV.

Mean±SD				

Hepatic cord distension	Hemorrhage in sinusoid	Vacuolar degenerations of hepatocytes	Total histopathological score	IHC score
0.2±0.45	0.8±0.45	2.6±0.55	1.2±1.25	1.5±1.78
0.8±0.45	0.8±0.84	0.2±0.45	0.6±0.35	0.3±0.48
1±0	0.6±0.55	1.2±0.45	0.9±0.3	0.5±0.53
1.4±0.55	0.4±0.55	0.4±0.55	0.7±0.57	0.9±0.99
1.4±0.55	0.4±0.55	0.6±0.55	0.8±0.53	0.6±0.69

SD: Standard deviation, IHC: Immunohistochemistry, PPV: *Porcine parvovirus*

**Table-2:** Overall histopathological scoring and the IHC scoring of uterus positive for PPV.

Mean±SD			

Perivascular cell infiltration in endometrium	Focal infiltration of cells in myometrium	Total histopathological score	Uterus IHC score (mean±SD)
0.2±0.45	0.8±0.45	0.5±0.42	0.3±0.48
1.8±0.45	1.2±0.45	1.5±0.42	0.5±0.71
0.8±0.45	1.2±0.45	1±0.28	0.4±0.52

SD: Standard deviation, PPV: *Porcine parvovirus*, IHC: Immunohistochemistry

## Conclusion

Diagnosis of PPV infection could be done with multiple approaches like serology in live animals and immunopathology in aborted fetus and placenta of sow. In this study, preliminary studies were carried out by ELISA and in future advance molecular studies will be carried out. Thus, seroprevalence study helps to detect the presence of the viral antibodies in a specific population and forms the basis to carry out a more comprehensive study on any infectious agent. IHC is the sensitive technique for diagnosis of PPV associated with the reproductive tract of swine and was found to supplement the gross and histopathological alterations, respectively, associated with the disease. Large-scale studies are required for detection and control of the disease.

## Authors’ Contributions

VM and NDS conceptualized the aim of the study, designed, planned, and supervised the experiments and corrected the manuscript. AK and PB collected the samples. AK, PB, GDL and GF performed IHC and ELISA. AK, GDL and NDS drafted the manuscript. HSB provided conceptual support and critically reviewed the manuscript. All authors read and approved the final manuscript.
